# Differential diagnosis of solid pancreatic lesions using contrast-enhanced three-dimensional ultrasonography

**DOI:** 10.1007/s00261-014-0135-8

**Published:** 2014-04-08

**Authors:** Haruo Miwa, Kazushi Numata, Kazuya Sugimori, Takashi Kaneko, Kentaro Sakamaki, Michio Ueda, Hiroyuki Fukuda, Katsuaki Tanaka, Shin Maeda

**Affiliations:** 1 Gastroenterological Center, Yokohama City University Medical Center, 4-57 Urafune-cho, Minami-ku, Yokohama, Kanagawa 232-0024 Japan; 2 Department of Biostatistics and Epidemiology, Yokohama City University Medical Center, 4-57 Urafune-cho, Minami-ku, Yokohama, Kanagawa 232-0024 Japan; 3 Division of Gastroenterology, Yokohama City University Graduate School of Medicine, 3-9 Fukuura, Kanazawa-ku, Yokohama, Kanagawa 236-0004 Japan

**Keywords:** Contrast-enhanced ultrasonography, Three-dimensional ultrasonography, Solid pancreatic lesion, Pancreatic ductal adenocarcinoma

## Abstract

**Purpose:**

To investigate the usefulness of contrast-enhanced three-dimensional ultrasonography (CE 3D US) for differential diagnosis of solid pancreatic lesions.

**Methods:**

Eighty-five patients with solid pancreatic lesions who underwent CE 3D US were retrospectively analyzed. Sixty-four patients had pancreatic ductal adenocarcinoma (PDAC), 10 had mass-forming pancreatitis (MFP), and 11 had neuroendocrine tumor (NET). Two blinded readers evaluated the enhancement patterns using four features: vascularity in the arterial phase, vascularity in the venous phase, vessel location, and vessel form. Vascularity in both phases was classified as hypervascular, isovascular, or hypovascular. Vessel location was classified into peritumoral or intratumoral. Vessel form was classified into fine or irregular. Kappa values were used to assess inter-reader agreement. The institutional review board approved this study, and informed consent was obtained.

**Results:**

Kappa values of the four features were 0.75, 0.72, 0.85, and 0.65, which were graded as good or excellent. The most typical combined enhancement pattern in PDAC was hypovascularity in both phases with peritumoral and irregular vessels; MFP was isovascular in both phases with intratumoral and fine vessels; and NETs were hypervascular in both phases with intratumoral and irregular vessels. The sensitivity and positive predictive value of the three patterns were 93.8% and 96.7% for the PDAC pattern, 80.0% and 100% for the MFP pattern, and 81.8%, and 69.2% for the NET pattern, respectively. The accuracy of these diagnostic criteria was 90.5%.

**Conclusion:**

CE 3D US allows detailed visualization of the enhancement patterns of various pancreatic lesions and can be used for the differential diagnosis.

A conventional ultrasonography (US) is first performed for the evaluation of solid pancreatic lesions [1–3]. However, this method alone cannot be used for the differential diagnosis of solid pancreatic lesions because most of these lesions are similarly hypoechoic on B-mode US [4, 5]. Doppler US is widely used to evaluate the relatively large vessels around the pancreas [6–8]; however, it cannot clearly reveal the vessels in solid pancreatic lesions.

Recently, contrast-enhanced US (CE US) has become increasingly important for the characterization of solid pancreatic lesions because it provides real-time scanning compared with contrast-enhanced computed tomography (CT) or magnetic resonance imaging (MRI) [9, 10]. Sonazoid (Daiichi Sankyo, Tokyo, Japan) is a second-generation contrast agent comprising perfluorobutane-based microbubbles (median diameter, 2–3 μm) that are stabilized by a phospholipid monolayer shell [11, 12]. Sonazoid permits repetitive scanning and provides precise enhanced images. Although several studies have indicated the importance of transabdominal CE US using Levovist (a first-generation contrast agent comprising galactose–palmitic acid; Shering AG, Berlin, Germany) or SonoVue (a second-generation contrast agent comprising sulfur hexafluoride-filled microbubbles with a phospholipid shell; Bracco Inc, Milan, Italy) for the characterization of pancreatic diseases, no study has reported the use of Sonazoid [13–21].

Sonazoid-enhanced US usually use a low mechanical index (MI) contrast mode [11]. In the case of solid pancreatic lesions, a low MI contrast mode setting does not destroy the microbubbles and enables the continuous observation of tumor enhancement. However, this mode cannot eliminate the background B-mode sufficiently, which makes it difficult to clearly evaluate minute vessels. In contrast, a high MI contrast mode setting can eliminate the background B-mode and provide sensitive visualization of tumor vessels [22–24].

Three-dimensional US (3D US) images provide more spatial information compared with two-dimensional (2D) US images [22, 25, 27]. With advances in 3D US technology, volume data can be acquired simply and rapidly with the automatic scan function using a mechanically swept curved array. Tomographic ultrasound images (TUIs) are reconstructed by dividing the images obtained from the volume data into multiple parallel slices in three orthogonal planes, which is similar to that in CT and MRI. In addition, sonographic angiograms similar to radiographic angiographic images are reconstructed for visualizing tumor vessels in three dimensions [24]. A combination of TUIs and sonographic angiograms can be used to simultaneously evaluate the vascularity in solid pancreatic lesions and the pancreatic parenchyma, providing a clear 3D view of the tumor vessels.

The aim of the present study was to investigate the usefulness of CE 3D US for the differential diagnosis of solid pancreatic lesions.

## Materials and methods

### Patients

Between February 2007 and March 2012, 85 consecutive patients with solid pancreatic lesions were retrospectively analyzed, including 64 patients with pancreatic ductal adenocarcinoma (PDAC), 10 with mass-forming pancreatitis (MFP), and 11 with neuroendocrine tumor (NET). All lesions could be detected by conventional US, and all patients underwent CE 3D US at the Yokohama City University Medical Center. After the CE 3D US examinations, pathological analyses were performed to achieve definitive diagnoses. Twenty-two patients were treated surgically, and in the remaining 63 patients, diagnosis was performed by analysis of the biopsy specimen obtained by endosonographic fine-needle aspiration biopsy (*n* = 45), endoscopic biopsy (*n* = 11), or percutaneous biopsy (*n* = 7). MFP was also confirmed by its typical appearance on multi-detector CT for at least 1 year during the follow-up period. The institutional review board approved this study, and informed that consent was obtained from all patients before the CE 3D US examinations were conducted.

### Equipment and contrast agent

The examinations were performed using a LOGIQ 7 ultrasound imaging system and a mechanically swept curved array (GE Healthcare, Milwaukee, WI) with a frequency of 2.0–5.5 MHz. Two sonographers (K.N. and H.M.) with 10 and 3 years of experience in abdominal CE US, respectively, performed these examinations. The coded harmonic angio mode, a high MI contrast mode (MI = 0.6–0.9) at 8–13 frames/s, was used, and the focal zone was set beneath the solid pancreatic lesions. All patients received an intravenous bolus injection of 0.2 mL of Sonazoid via the antecubital vein, followed by infusion of a 5 mL 5% glucose solution prior to the automatic sweeping of the transducer.

The autosweep 3D function was used for image acquisition in two phases: 3–4 times in the arterial phase (10–60 s after injection), and 2 times in the venous phase (90–180 s after injection). A volume angle of between 40° and 80° (mean, 57°) was selected to simultaneously acquire images of focal pancreatic lesions and pancreatic parenchyma. The acquisition time was 1.3 s for a volume angle of 40° and 3.2 s for a volume angle of 80°. Acquisition was performed in various directions to avoid artifacts such as gastrointestinal gas, major vessels, and calcification around the pancreas.

### Image reconstruction

**Fig. 1:**
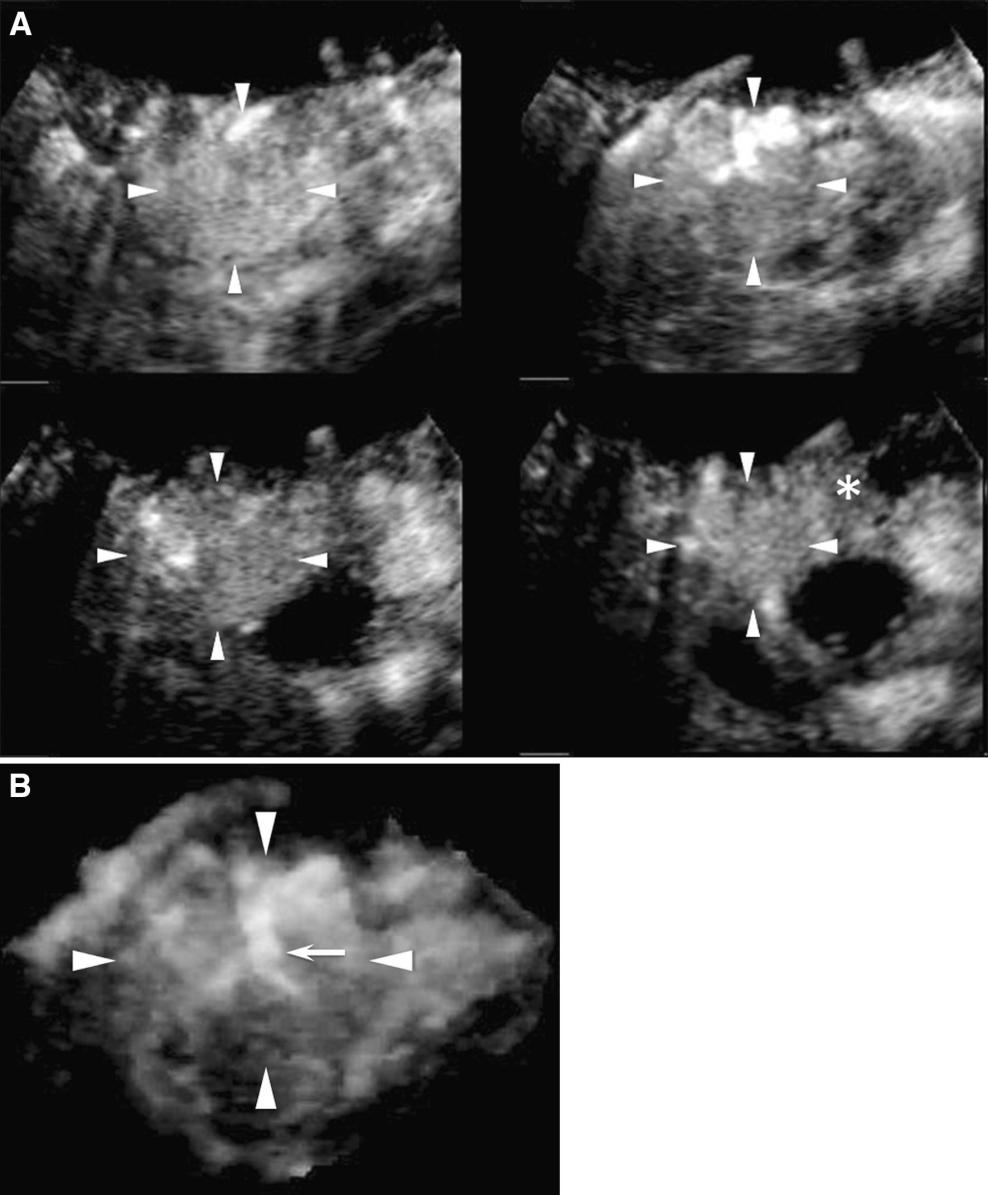
CE 3D US images acquired in the transverse plane of a 71-year-old woman with NET (maximum diameter, 38 mm) in the pancreatic head. **A** TUIs of CE 3D US (plane A) in the arterial phase show a hypervascular lesion (*arrowheads*) compared with the parenchyma in the pancreatic body (*asterisk*). **B** Sonographic angiogram in the arterial phase, rendered in the maximum intensity mode, shows a markedly dilated tortuous vessel, classified as an intratumoral and irregular vessel (*arrow*).

**Fig. 2:**
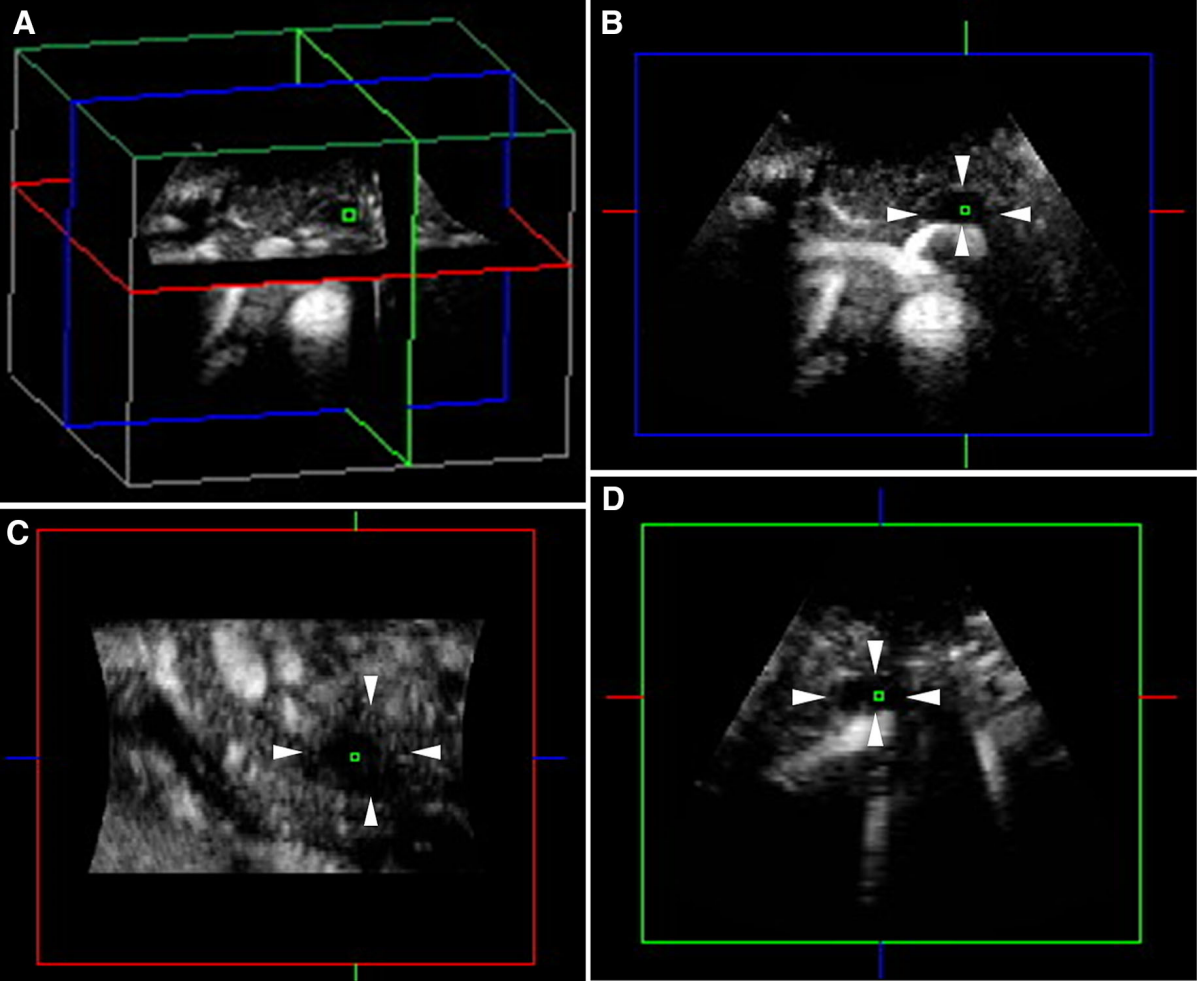

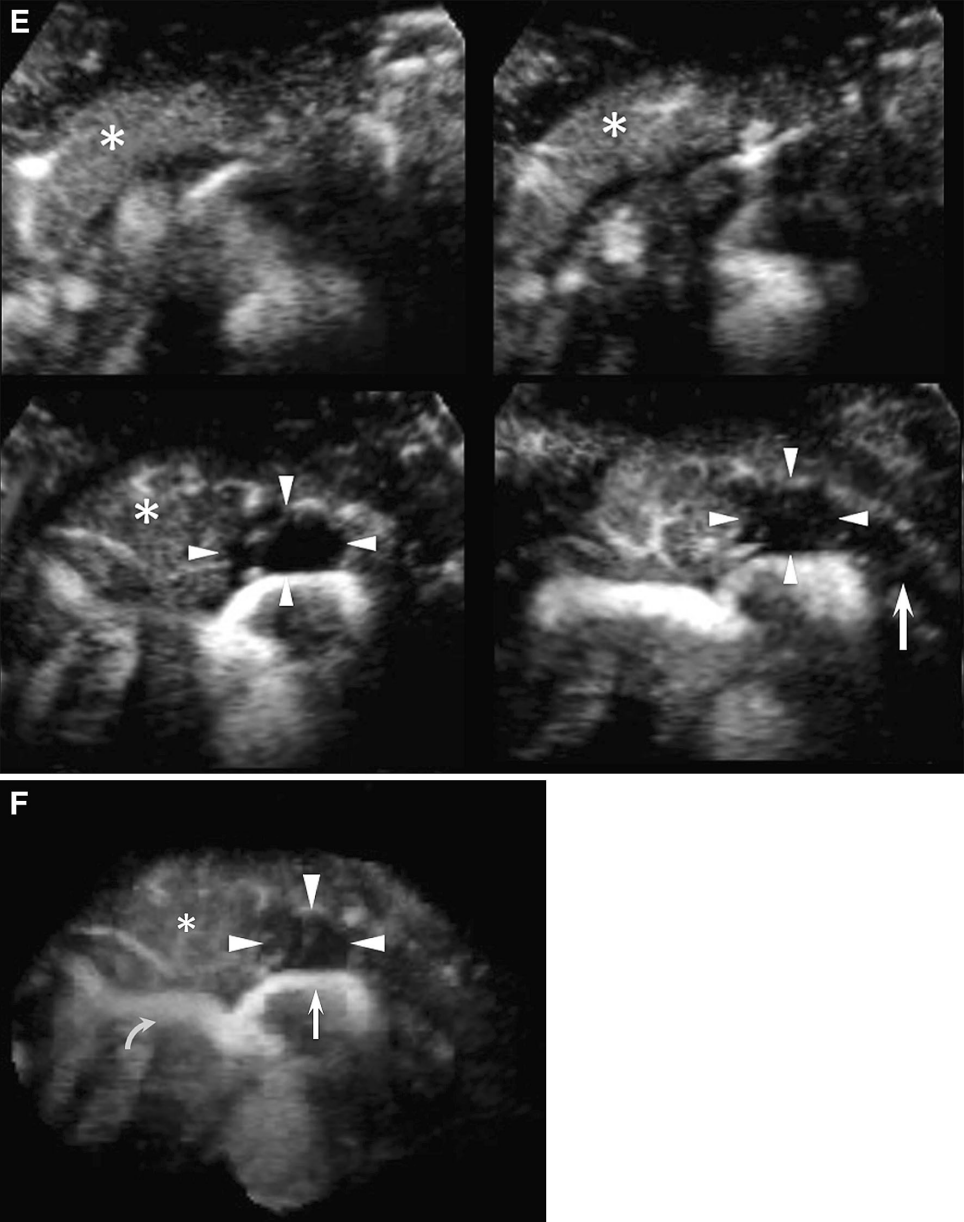
CE 3D US images acquired in the transverse plane of a 43-year-old man with PDAC (maximum diameter, 38 mm) in the pancreatic body. **A**–**D** Hypovascular lesions (*arrowheads*) are clearly seen in three orthogonal planes in the arterial phase. *Small green squares* show the center of a lesion. (**A** An image for navigation of the following planes; **B** plane A, from the front to the back; **C** plane B, from the left to the right; and **D** plane C, from the top to the bottom). **E** TUIs of CE 3D US (plane A) in the arterial phase show a hypovascular lesion (*arrowheads*) compared with the parenchyma in the pancreatic head (*asterisks*) with peritumoral and fine vessels. The dilated main pancreatic duct appears as a non-enhanced area in the caudal part of the lesion (*arrow*). **F** Sonographic angiogram in the arterial phase, rendered in the average intensity mode, shows the hypovascular lesion in the pancreatic body (*arrowheads*) compared with the parenchyma (*asterisk*). The splenic artery with encasement by tumor invasion (*arrow*) and common hepatic artery (*curved arrow*) is clearly seen posterior to the pancreas.

**Fig. 3:**
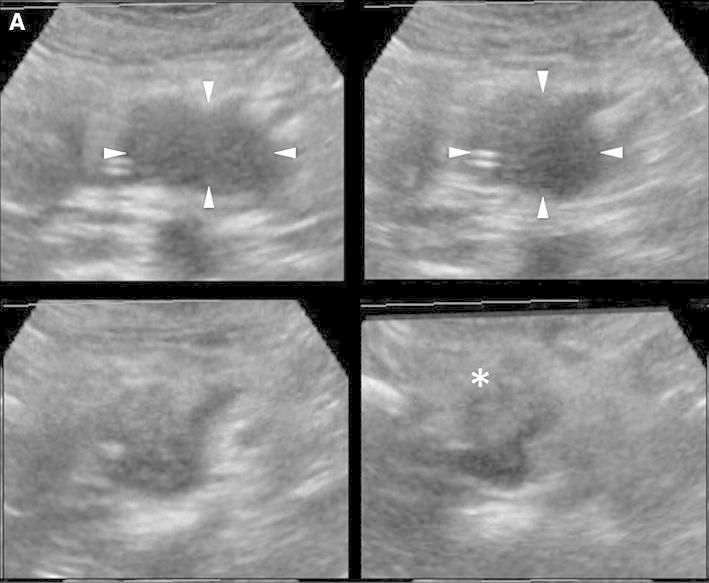

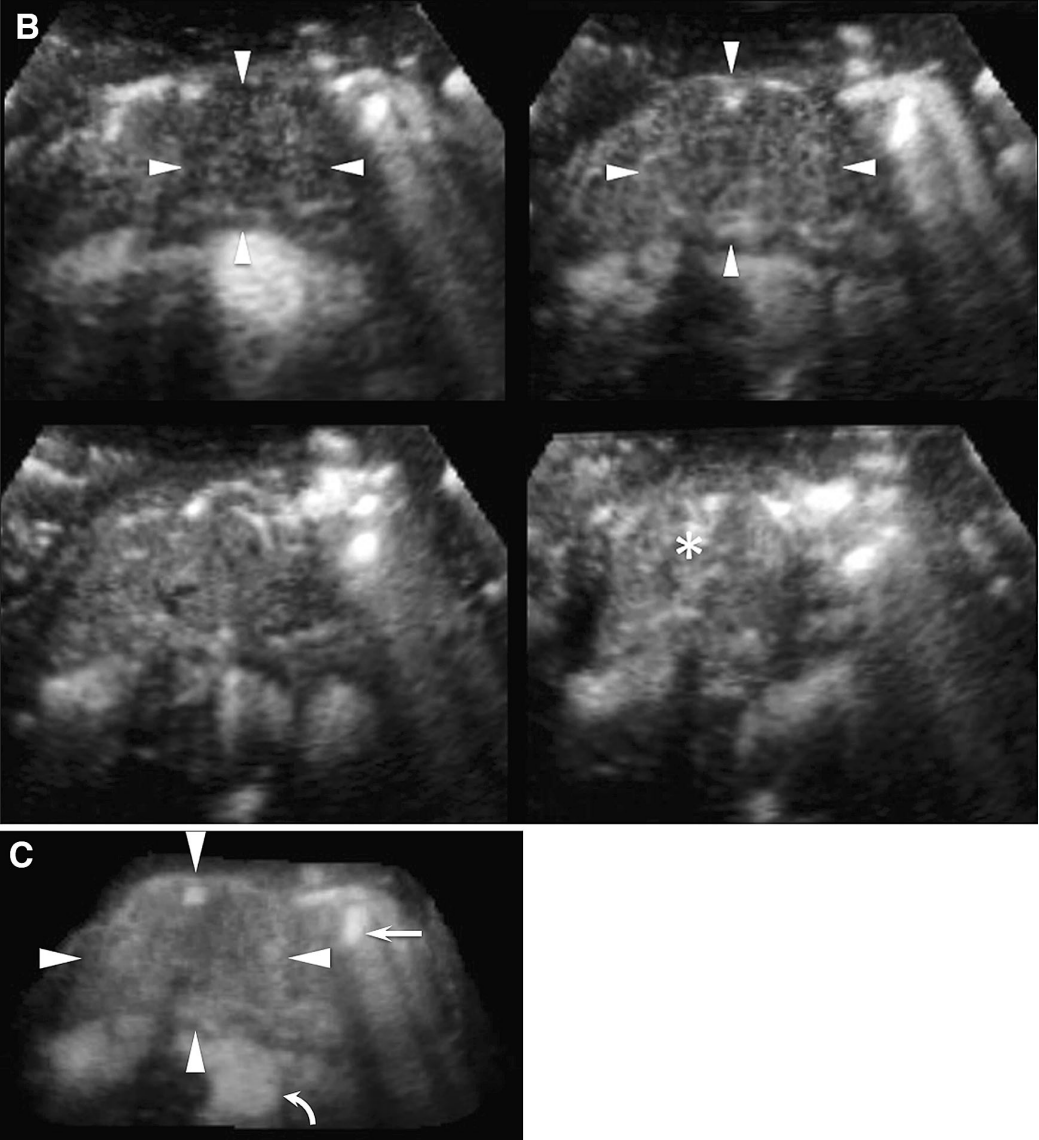
Conventional 3D US and CE 3D US images acquired in the transverse plane of a 78-year-old woman with MFP (maximum diameter, 33 mm) in the pancreatic head. **A** TUI of 3D US (plane A) in the B-mode shows a hypoechoic lesion in the pancreatic head (*arrowheads*). The image on the lower right shows the pancreatic parenchyma in the pancreatic body (*asterisk*). **B** TUIs of CE 3D US (plane A) in the arterial phase show an isovascular lesion (*arrowheads*) compared with the pancreatic parenchyma (*asterisk*). Dense fine vessels are seen in a whole lesion, hypoechoic on B-mode US, similar to the pancreatic parenchyma. **C** Sonographic angiogram in the arterial phase, rendered in the average intensity mode, shows homogeneous enhancement (*arrowheads*) of both the pancreatic head and body. Superior mesenteric artery (*arrow*) and abdominal aorta (*curved arrow*) appear around pancreas.

After volume data acquisition, we could check the CE 3D US images frame by frame, similar to the 2D image. Then, the best images were selected for 3D image reconstruction, which was performed using an in-built function of the LOGIQ 7 ultrasound system. The volume of interest was depicted as a cuboid region in which the three orthogonal imaging planes were reconstructed: from the front to the back through the cuboid region, defined as plane A; from the left to the right, defined as plane B; and from the top to the bottom, defined as plane C [24]. The time required to perform this procedure was approximately 1 min. TUIs in each plane were reconstructed by dividing the images obtained from the volume data into four or six parallel slices, so that the solid pancreatic lesions and pancreatic parenchyma could be observed simultaneously. The distance between any two slices was approximately 2–10 mm. The time required to perform this procedure was approximately 20 s for plane A and 30 s for plane B or C. Sonographic angiograms were reconstructed by two kinds of rendering modes that allowed 3D visualization of the vessels and enhancement of solid pancreatic lesions. The maximum intensity mode to display maximal gray values in the volume of interest was used to visualize vessels in solid pancreatic lesions (Fig. 1B). The average intensity mode to display average gray values in the volume of interest was used to compare vascularity of a solid pancreatic lesion and pancreatic parenchyma (Figs. 2F, 3C). The mean time required for this procedure was approximately 1 min. Total time for 3D image reconstruction was no more than 5 min for each lesion. The volume data, TUIs, and sonographic angiograms were stored electronically.

### Image evaluation

**Fig. 4:**
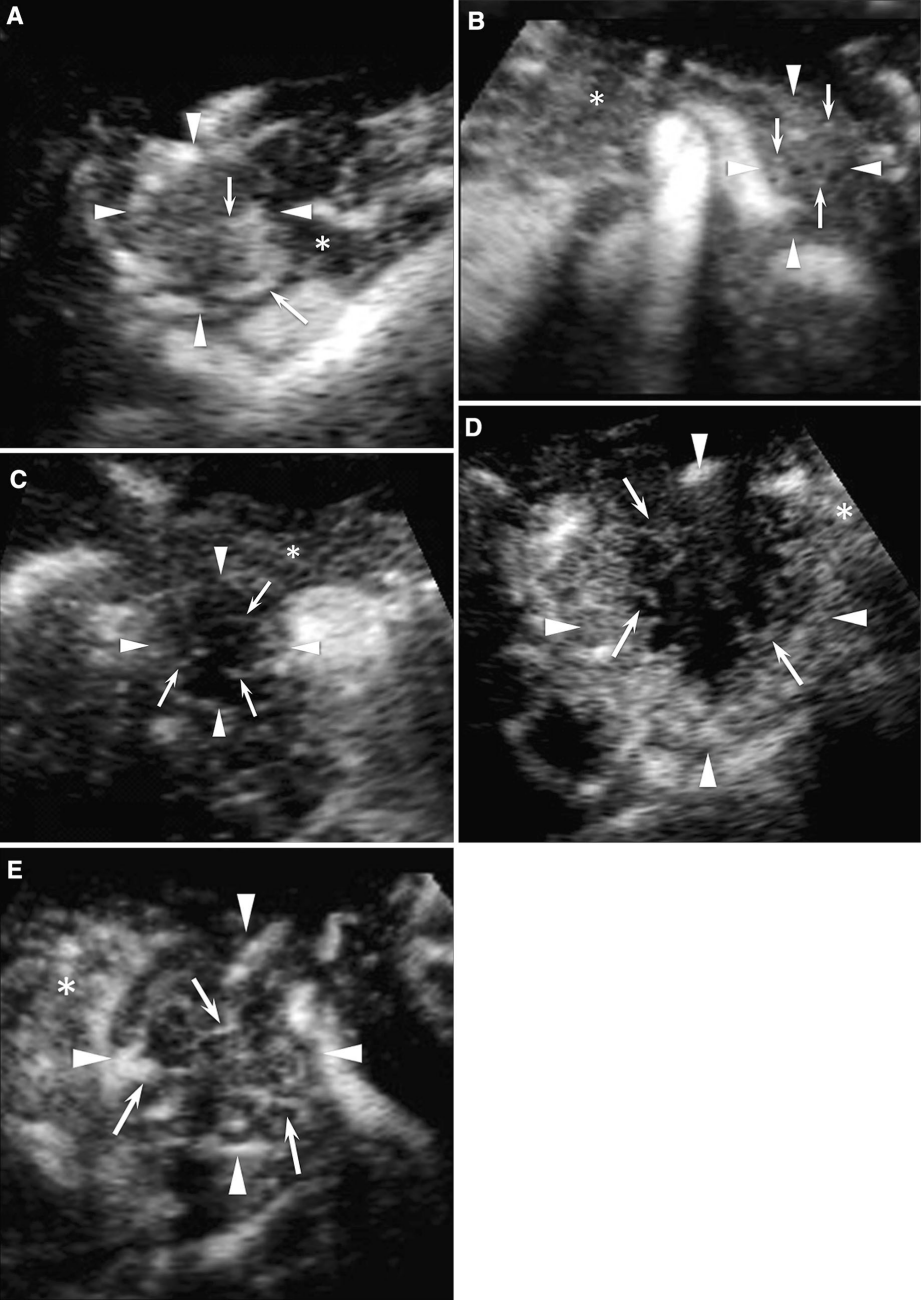
CE 3D US images showing the typical enhancement patterns of solid pancreatic lesions. **A** A hypervascular lesion (*arrowheads*) compared with the pancreatic parenchyma (*asterisk*) with intratumoral and irregular vessels (*arrows*). **B** An isovascular lesion (*arrowheads*) compared with the pancreatic parenchyma (*asterisk*) with intratumoral and fine vessels (*arrows*). **C** A hypovascular lesion (*arrowheads*) compared with the pancreatic parenchyma (*asterisk*) with peritumoral and fine vessels (*arrows*). **D** A hypovascular lesion (*arrowheads*) compared with the pancreatic parenchyma (*asterisk*) with peritumoral and irregular vessels (*arrows*). **E** Hypovascular lesions (*arrowheads*) compared with the pancreatic parenchyma (*asterisk*) with intratumoral and irregular vessels (*arrows*).

After all the examinations were completed, the two above-mentioned sonographers defined the evaluation criteria (Fig. 4). Vascularity in the arterial and venous phases was classified as follows: hypervascular, higher echogenicity than that of the pancreatic parenchyma; isovascular, equal echogenicity to that of the pancreatic parenchyma; and hypovascular, lower echogenicity than that of the pancreatic parenchyma. Vessel patterns were characterized by vessel location and form. Vessels located only at the peripheral area of the focal lesions were termed “peritumoral vessels” and those located at both the peripheral and central areas were termed “intratumoral vessels.” With regard to the vessel form, “fine vessels” were thin, minute, and orderly branched, while “irregular vessels” were winding, tortuous, and variably shaped. Two readers (K.S. and H.F.) with 5 and 3 years of experience in CE 3D US imaging of the pancreas, blinded to the final diagnosis and other radiological information, independently interpreted all the images according to the evaluation criteria, respectively. If the readers obtained discordant results, then they arrived at the final classification after a consensus meeting. After pattern classification, the combination of enhancement patterns was summarized.

### Statistical analysis

Kappa values were used to assess inter-reader agreement regarding the characteristic enhancement patterns of solid pancreatic lesions in each classification before consensus meeting. Inter-reader agreement was graded as poor (<0.20), moderate (0.20–0.40), fair (0.40–0.60), good (0.60–0.80), or excellent (0.80–1.00). The enhancement patterns, vascularity in the arterial and venous phases, vessel location, and vessel form were used to establish the diagnostic criteria for solid pancreatic lesions. We calculated the positive predictive value (PPV) of PDAC, MFP, and NET for each of the nine combination patterns and defined them as the PDAC, MFP, and NET patterns, according to the largest PPV. Next, the sensitivity and PPV for differential diagnosis of each pattern-based classification were calculated. Accuracy was defined by the percentage of patients who were diagnosed correctly. All statistical analyses were performed using SPSS software (version 19; SPSS, Tokyo, Japan).

## Results

### Patient characteristics

Characteristics of the patients included in this study are summarized in Table 1. The patients consisted of 47 men and 38 women. The mean age of the patients was 68 years (range, 41–83 years). In total, 57 of the lesions in these patients were located in the pancreatic head and 28 were located in the body and tail. The size of the lesions ranged from 10 to 62 mm (average, 28 mm) in diameter on B-mode US.

**Table 1:** Clinical characteristics of the subjects with solid pancreatic lesions enrolled in this study

Characteristics	
No. of patients (PDAC/MFP/NET)	85 (64/10/11)
Age (mean, range, years)	68, 41–83
Sex (male/female)	47/38
Location of lesions (pancreatic head/pancreatic body and tail)	57/28
Diameter of lesions (mean, range, mm)	28, 10–62
Diagnosis confirmed by surgery/biopsy	19/65

*PDAC* pancreatic ductal adenocarcinoma, *MFP* mass-forming pancreatitis, *NET* neuroendocrine tumor

## Enhancement patterns according to each reader

The enhancement patterns observed on CE 3D US by each of the two readers and the kappa values are summarized in Table 2. In 60 (71%) of the 85 cases, the readers obtained completely concordant results in each classification. In the remaining 25 (29%) cases, discordant results were obtained; more specifically, results related to the vascularity in the arterial phase and the venous phase, vessel location, and vessel form were discordant in 8 (9%), 9 (11%), 5 (6%), and 10 (12%) of the 85 cases, respectively. The kappa values of vascularity in the arterial and venous phases, vessel location, and vessel form were 0.75, 0.72, 0.85, and 0.65, respectively. They were graded as good or excellent.

**Table 2:** Enhancement patterns

	Reader 1			Reader 2			Kappa value
	PDAC	MFP	NET	PDAC	MFP	NET	
**Arterial phase**							0.75
Hypervascular	3(2/64)	0(0/10)	73(8/11)	3(2/64)	0(0/10)	64(7/11)	
Isovascular	10(7/64)	100(10/10)	27(3/11)	14(9/64)	90(9/10)	36(4/11)	
Hypovascular	86(55/64)	0(0/10)	0(0/11)	83(53/64)	10(1/10)	0(0/11)	
**Venous phase**							0.72
Hypervascular	2(1/64)	0(0/10)	64(7/11)	2(1/64)	0(0/10)	64(7/11)	
Isovascular	2(1/64)	90(9/10)	36(4/11)	6(4/64)	80(8/10)	27(3/11)	
Hypovascular	97(62/64)	10(1/10)	0(0/11)	83(53/64)	20(2/10)	9(1/11)	
**Vessel location**							0.85
Peritumoral	52(33/64)	0(0/10)	0(0/11)	52(33/64)	0(0/10)	0(0/11)	
Intratumoral	48(31/64)	100(10/10)	100(11/11)	48(31/64)	100(10/10)	100(11/11)	
**Vessel form**							0.65
Fine	16(10/64)	70(7/10)	0(0/11)	16(10/64)	90(9/10)	0(0/11)	
Irregular	84(54/64)	30(3/10)	100(11/11)	84(54/64)	10(1/10)	100(11/11)	

Data are percentages; data in parentheses are numbers of the solid pancreatic lesions

*PDAC* pancreatic ductal adenocarcinoma, *MFP* mass-forming pancreatitis, *NET* neuroendocrine tumor

## Final enhancement patterns

In the 25 cases where the readers obtained discordant results, they arrived at the final classification after a consensus meeting. The enhancement patterns agreed upon after the consensus meeting are shown in Table 3.

**Table 3:** Enhancement patterns after consensus meeting

	PDAC	MFP	NET
**Arterial phase**			
Hypervascular	3(2/64)	0(0/10)	64(7/11)
Isovascular	13(8/64)	90(9/10)	36(4/11)
Hypovascular	84(54/64)	10(1/10)	0(0/11)
**Venous phase**			
Hypervascular	2(1/64)	0(0/10)	55(6/11)
Isovascular	5(3/64)	90(9/10)	36(4/11)
Hypovascular	94(60/64)	10(1/10)	9(1/11)
**Vessel location**			
Peritumoral	48(31/64)	0(0/10)	0(0/11)
Intratumoral	52(33/64)	100(0/10)	100(11/11)
**Vessel form**			
Fine	16(10/64)	90(9/10)	0(0/11)
Irregular	84(54/64)	10(1/10)	100(11/11)

Data are percentages; data in parentheses are numbers of the solid pancreatic lesions

*PDAC* pancreatic ductal adenocarcinoma, *MFP* mass-forming pancreatitis, *NET* neuroendocrine tumor

### Vascularity

In the arterial phase, 54 (84%) of the 64 PDACs were hypovascular; 9 (90%) of the 10 MFP were isovascular; and 7 (64%) of the 11 NETs were hypervascular. In the venous phase, 60 (94%) of the 64 PDACs were hypovascular; 9 (90%) of the 10 MFP were isovascular; and 6 (55%) and 4 (36%) of the 11 NETs were hypervascular and isovascular, respectively. One (50%) of the 2 PDACs that were hypervascular in the arterial phase changed to isovascular in the venous phase. Six (75%) of the 8 PDACs that were isovascular in the arterial phase changed to hypovascular in the venous phase. These “washout changes” were a characteristic finding for PDACs; in contrast, these changes were not found in MFP and in 9 (82%) of the 11 NETs.

### Vessel patterns

Regarding the vessel location, 31 (48%) and 33 (52%) of the 64 PDACs were located in the peritumoral and intratumoral vessels, respectively. In contrast, all MFP and NET lesions were located in the intratumoral vessels. Regarding the vessel form, of the 64 PDAC patients, 25 (39%) had fine vessels and 39 (61%) had irregular vessels. Nine (90%) of the 10 MFP patients had fine vessels. In contrast, all NET patients had irregular vessels.

### Combinations of enhancement patterns

Combinations of enhancement patterns are summarized in Table 4. These patterns were characterized by four features: vascularity in the arterial phase, vascularity in the venous phase, vessel location, and vessel form. A total of 36 combinations are possible. However, all the lesions were classified into nine patterns in the present study. PPV was calculated from the results, and each combination was defined as a PDAC, MFP, or NET pattern, according to the largest PPV. Sixty-two lesions showing patterns 5, 6, 7, 8, and 9 were defined as the PDAC pattern. The most typical pattern was hypovascular in both the phases with peritumoral irregular vessels. One case of MFP and one case of NET demonstrated the PDAC pattern. Eight lesions showing pattern 3 were defined as the MFP pattern. This pattern was isovascular in both the phases with intratumoral fine vessels. Neither PDAC nor NET lesion demonstrated the MFP pattern. Thirteen lesions showing patterns 1 and 4 were defined as the NET pattern. The most typical pattern was hypervascular in both the phases with intratumoral irregular vessels. Three PDAC lesions and one MFP lesion demonstrated the NET patterns. Two lesions showing pattern 2 could not be defined because PPVs for NETs and PDACs were equal.

**Table 4:** Combinations of enhancement patterns

No.	Combinations of enhancement patterns				Positive predictive value			Diagnostic pattern
	Arterial phase	Venous phase	Vessel location	Vessel form	PDAC	MFP	NET	
1	Hypervascular	Hypervascular	Intratumoral	Irregular	0.14 (1)		0.86 (6)	NET pattern
2	Hypervascular	Isovascular	Intratumoral	Irregular	0.50 (1)		0.50 (1)	Undefined
3	Isovascular	Isovascular	Intratumoral	Fine		1.00 (8)		MFP pattern
4	Isovascular	Isovascular	Intratumoral	Irregular	0.33 (2)	0.17 (1)	0.50 (3)	NET pattern
5	Isovascular	Hypovascular	Intratumoral	Fine	0.86 (6)		0.14 (1)	PDAC pattern
6	Hypovascular	Hypovascular	Peritumoral	Fine	1.00 (8)			PDAC pattern
7	Hypovascular	Hypovascular	Peritumoral	Irregular	1.00 (23)			PDAC pattern
8	Hypovascular	Hypovascular	Intratumoral	Fine	0.67 (2)	0.33 (1)		PDAC pattern
9	Hypovascular	Hypovascular	Intratumoral	Irregular	1.00 (21)			PDAC pattern

Data in parentheses are numbers of the solid pancreatic lesions

*PDAC* pancreatic ductal adenocarcinoma, *MFP* mass-forming pancreatitis, *NET* neuroendocrine tumor

For the final pathological diagnosis, the sensitivity and PPV for each of the three patterns, on the basis of the combined enhancement patterns, were 93.8% [95% confidence interval (CI) 89.1–98.7%] and 96.7% (95% CI 95.2–100%) for the PDAC pattern; 80.0% (95% CI 70.0–100%) and 100% (95% CI 100–100%) for the MFP pattern; and 81.8% (95% CI 72.7–100%) and 69.2% (95% CI 53.8–97.6%) for the NET pattern, respectively. In total, 77 of the 85 cases, which included 60 of the 64 PDACs, 8 of the 10 MFP, and 9 of the 11 NETs, were correctly diagnosed using the combination-based approach. Accordingly, the accuracy of this diagnostic method was 90.5% (95% CI 90.4–90.8%).

## Discussion

There are no published reports regarding the diagnosis of pancreatic disease using CE 3D US with Sonazoid. According to our experience, the vessels in the pancreas are detectable from approximately 10 s after infusion, and the pancreatic parenchyma is rapidly enhanced until approximately 30 s. Subsequently, the enhancement of the pancreatic parenchyma persists until approximately 60 s and then gradually reduces from 90 to 180 s. Based on these features, we have defined two phases, i.e., the arterial phase of 10–60 s and the venous phase of 90–180 s. With respect to differences from other contrast agents, Sonazoid is longer acting than Levovist [15, 16, 22–24]. This feature enables repetitive autosweep acquisitions in the arterial phase, which is essential for CE 3D US. Moreover, with Sonazoid, we can use high MI contrast mode settings that provide precise CE 3D US images. In contrast, this mode is not available for SonoVue [17–20].

In this study, CE 3D US was used to simultaneously evaluate solid pancreatic lesions and pancreatic parenchyma. Moreover, the combinations of enhancement patterns obtained using CE 3D US revealed the characteristic features of specific solid pancreatic lesions such as PDACs, MFP, and NETs, which enabled their differential diagnosis.

Several studies have attempted to characterize solid pancreatic lesions using CE US. D’Onofrio et al. [21] reported that 891 (90%) of the 987 PDACs were hypovascular on SonoVue-enhanced US, and this finding was useful for the differential diagnosis of solid pancreatic lesions. Sofuni et al. [16] reported that 34 (87%) of the 39 PDACs were hypovascular on Levovist-enhanced US. However, these reports also suggested that approximately 10% of PDACs showed isovascularity or hypervascularity and could be easily misdiagnosed. Matsubara et al. reported a time-intensity curve-based quantitative analysis of CE harmonic endosonographic ultrasound (EUS) for differentiating pancreatic diseases. They reported that the echo-intensity reduction rate from the peak to 1 min after Sonazoid injection was greater in PDACs than in MFP and NETs [28]. In the present study, vascularity of PDACs also decreased from the arterial phase to the venous phase. We termed this transition in vascularity as a “washout change,” which refers to the change from hypervascular lesions in the arterial phase to isovascular or hypovascular lesions in the venous phase or from isovascular lesions in the arterial phase to hypovascular lesions in the venous phase. In the present study, 7 (70%) of the 10 PDACs that were hypervascular of isovascular in the arterial phase showed washout changes in the venous phase. In contrast, all 10 cases of MFP and 9 (82%) of the 11 NETs showed persistent enhancement during both the phases. As an exception, 3 (5%) of the 64 PDACs showed persistent enhancement and could not be differentiated from MFP and NETs. In one PDAC case with persistent enhancement, pathological findings of the resected specimen revealed a feature with neuroendocrine differentiation, and in the other two cases of PDAC, severe inflammation was observed in the tumors.

In solid pancreatic lesions, vessel form is an important feature for the differentiation of MFP and NETs. Nine (90%) of the 10 MFP cases showed fine vessels, similar to pancreatic parenchyma, whereas all the NET cases showed irregular vessels. The irregular vessels in NETs were markedly dilated compared with those in PDACs and MFP, with the exception of one (10%) MFP case that showed irregular vessels, possibly because of severe inflammation in the lesion. Regarding the vessel location, peritumoral vessels were unique in PDAC cases. Finally, nine combined enhancement patterns were consensually obtained by the two readers. According to PPV, these combination patterns were classified as a PDAC, MFP, or NET pattern. These combination patterns enabled us to perform a highly accurate differential diagnosis of solid pancreatic tumors.

Several recent studies have suggested the usefulness of CE harmonic EUS in the diagnosis of pancreatic disease [28–31]. Compared with transabdominal US, EUS has the advantages of higher spatial resolution and reduction of artifacts from abdominal gas. However, the viewing angle of EUS is smaller than that of transabdominal US; therefore, it is often difficult to simultaneously depict solid pancreatic lesions and pancreatic parenchyma.

Similar to 2D images, 3D US images enable us to repeatedly check the acquired volume data frame by frame. Moreover, CE 3D US has the advantages of a wider volume angle and 3D viewing from various directions because of the automatic volume data acquisition setting. We can compare both the location and the vascularity of solid pancreatic lesions and those of pancreatic parenchyma simultaneously in a single reconstructed plane. These are helpful to determine the focal lesions in the pancreas by tracing the dilated main pancreatic duct or common bile duct and to discern the anatomical structures around the pancreas, such as the vessels or lymph nodes.

This study has several limitations. First, US of the pancreas include artifacts such as those from abdominal gas and motion, and calcification of the large vessels surrounding the pancreas. Furthermore, the pancreatic area behind the major vessels becomes hyper-echoic on CE US imaging in the high MI contrast mode. In particular, the artifacts behind the gastroduodenal artery, superior mesenteric artery, and superior mesenteric vein may hinder the accurate evaluation of lesions in the pancreatic head. Second, the number of MFP and NET cases was less than the number of PDAC cases because of the relatively low prevalence of MFP and NETs. This may have biased the results of the present study; therefore, in the future, these results should be confirmed using a larger number of cases. Third, the diagnosis criteria reported in this study were not applied prospectively. Therefore, a prospective study using the enhancement patterns-based classification described in this study is needed to confirm its accuracy for the differential diagnosis of solid pancreatic lesions.

In summary, we classified solid pancreatic lesions into the combinations of enhancement patterns using CE 3D US. We believe that this modality has clinical benefits for the accurate differential diagnosis of solid pancreatic lesions.

## References

[1] Minniti S, Bruno C, Biasiutti C (2003). Sonography versus helical CT in identification and staging of pancreatic ductal adenocarcinoma. J Clin Ultrasound.

[2] Karlson BM, Ekbom A, Lindgren PG (1999). Abdominal US for diagnosis of pancreatic tumor: prospective cohort analysis. Radiology.

[3] Hohl C, Schmidt T, Haage P (2004). Phase-inversion tissue harmonic imaging compared with conventional B-mode ultrasound in the evaluation of pancreatic lesions. Eur Radiol.

[4] Yang W, Chen MH, Yan K (2007). Differential diagnosis of non-functional islet cell tumor and pancreatic carcinoma with sonography. Eur J Radiol.

[5] D’Onofrio M, Gallotti A, Pozzi Mucelli R (2010). Imaging techniques in pancreatic tumors. Expert Rev Med Devices.

[6] Tomiyama T, Ueno N, Tano S (1996). Assessment of arterial invasion in pancreatic cancer using color Doppler ultrasonography. Am J Gastroenterol.

[7] Rickes S, Unkrodt K, Neye H (2002). Differentiation of pancreatic tumors by conventional ultrasound, unenhanced and echo-enhanced power Doppler sonography. Scand J Gastroenterol.

[8] Bertolotto M, D’Onofrio M, Martone E (2007). Ultrasonography of the pancreas. 3. Doppler imaging. Abdom Imaging.

[9] Motosugi U, Ichikawa T, Morisaka H (2011). Detection of pancreatic carcinoma and liver metastases with gadoxetic acid-enhanced MR imaging: comparison with-contrast enhanced multi-detector row CT. Radiology.

[10] D’Onofrio M, Malagò R, Zamboni G (2005). Contrast-enhanced ultrasonography better identifies pancreatic tumor vascularization than helical CT. Pancreatology.

[11] Sontum PC (2008). Physicochemical characteristics of Sonazoid, a new contrast agent for ultrasound imaging. Ultrasound Med Biol.

[12] Watanabe R, Matsumura M, Chen CJ (2005). Characterization of tumor imaging with microbubble-based ultrasound contrast agent Sonazoid, in rabbit liver. Biol Pharm Bull.

[13] Kim T, Murakami T, Takamura M (2001). Pancreatic Mass due to chronic pancreatitis: correlation of CT and MR imaging features with pathologic findings. AJR Am J Roentgenol.

[14] Oshikawa O, Tanaka S, Ioka T (2002). Dynamic sonography of pancreatic tumors: comparison with dynamic CT. AJR Am J Roentgenol.

[15] Ozawa Y, Numata K, Tanaka K (2002). Contrast-enhanced sonography of small pancreatic mass lesion. J Ultrasound Med.

[16] Sofuni A, Iijima H, Moriyasu F (2005). Differential diagnosis of pancreatic tumors using ultrasound contrast imaging. J Gastroenterol.

[17] D’Onofrio M, Zamboni G, Tognolini A (2006). Mass-forming pancreatitis: value of contrast-enhanced ultrasonography. World J Gastroenterol.

[18] Dietrich CF, Braden B, Hocke M (2008). Improved characterization of solitary solid pancreatic tumours using contrast-enhanced transabdominal ultrasound. J Cancer Res Clin Oncol.

[19] Kersting S, Konopke R, Kersting F (2009). Quantitative perfusion analysis of transabdominal contrast-enhanced ultrasonography of pancreatic masses and carcinomas. Gastroenterology.

[20] Malagò R, D’Onofrio M, Zamboni GA (2009). Contrast-enhanced sonography of nonfunctioning pancreatic neuroendocrine tumors. AJR Am J Roentgenol.

[21] D’Onofrio M, Barbi E, Dietrich CF (2012). Pancreatic multicenter ultrasound study (PAMUS). Eur J Radiol.

[22] Luo W, Numata K, Morimoto M (2009). Clinical utility of contrast-enhanced three-dimensional ultrasound imaging with Sonazoid: findings on hepatocellular carcinoma lesions. Eur J Radiol.

[23] Luo W, Numata K, Morimoto M (2010). Differentiation of focal liver lesions using three-dimensional ultrasonography: retrospective and prospective studies. World J Gastroenterol.

[24] Luo W, Numata K, Morimoto M (2009). Focal liver tumors: characterization with 3D perflubutane microbubble contrast agent-enhanced US versus 3D contrast-enhanced multidetector CT. Radiology.

[25] Dietrich CF (2002). 3D real time contrast-enhanced ultrasonography, a new technique. Rofo.

[26] Wilson SR, Gupta C, Eliaziw M, Andrew A (2009). Volume imaging in the abdomen with ultrasound: how we do it. AJR Am J Roentgenol.

[27] Pezzilli R, Serra C, Calculli L (2013). Three-dimensional contrast-enhanced ultrasonography of intraductal papillary mucinous neoplasms of the pancreas: a comparison with magnetic resonance imaging. Pancreas.

[28] Matsubara H, Itoh A, Kawashima H (2011). Dynamic quantitative evaluation of contrast-enhanced endoscopic ultrasonography in the diagnosis of pancreatic diseases. Pancreas.

[29] Kitano M, Sakamoto H, Matsui U (2008). A novel perfusion imaging technique of the pancreas: contrast-enhanced harmonic EUS (with video). Gastrointest Endosc.

[30] Napoleon B, Alvarez-Sanchez MV, Gincoul R (2010). Contrast-enhanced harmonic endoscopic ultrasound in solid lesions of the pancreas: results of a pilot study. Endoscopy.

[31] Kitano M, Kudo M, Yamao K (2012). Characterization of small solid tumor in the pancreas: the value of contrast-enhanced harmonic endoscopic ultrasonography. Am J Gastroenterol.

